# Taking Us Out of Ourselves: Using Classroom Intergenerational Reading Groups to Connect Younger/Older Adults

**DOI:** 10.1093/geroni/igaf122.2507

**Published:** 2025-12-31

**Authors:** Marjorie Getz

**Affiliations:** Methodist College, Peoria, Illinois, United States

## Abstract

Communities become better connected when people from different ages and backgrounds become equals working together for a common goal. We report on a service-learning project focused on intergenerational reading of books related to successful aging, which brought people with different life perspectives together for shared educational experiences. Experiential learning projects are key for the gerontology certificate program at a midwestern nursing/health science college. All courses are focused on increased understanding of course content related to aging through project reflective practices, direct contact with older adults, exposure to themes related to community health and well-being, and an improved sense of community service/civic responsibility. The service-learning opportunities for each course (and the certificate curriculum itself) fit a hierarchy leading to increased competence and skills development when working with older adults. This project incorporated data from the reading group service-learning project between undergraduate students and members of a local OLLI program (ongoing since 2018). Forty-seven students (across time, interacting with 80 OLLI participants) provided reflective data about the project experience. Qualitative analyses were used; and content analyses of students’ reflections identified five themes: (a) communication skills development/enhancement (b) appreciation of older adults as vital community members; (c) processes involved in a successful reading group; (d) recognition of a variety of settings for learning opportunities in support of development as lifelong learners and for professional growth; and (e) appreciation of collaborative teamwork and expanded definition of peer. Other experiential learning activities in this joint nursing/gerontology curriculum are highlighted

